# Post-Coital Sudden Cardiac Arrest Due to Non-Traumatic Subarachnoid Hemorrhage—A Case Report

**DOI:** 10.21980/J8663N

**Published:** 2020-07-15

**Authors:** Vinson Vong, John Costumbrado, Daniel Ng, Brandon Phong

**Affiliations:** * Riverside Community Hospital/University of California Riverside, Department of Emergency Medicine, Riverside, CA; ^ University of California, Riverside School of Medicine, Riverside, CA

## Abstract

Emergency medicine (EM) learners are taught to approach cardiac arrest algorithmically using Advanced Cardiac Life Support (ACLS) with particular emphasis on treatment. However, when treating patients in cardiac arrest it is important to maintain a broad differential of possible non-cardiac etiologies of the presenting symptoms.

A patient presented to the emergency department (ED) as a post-coital cardiac arrest with prehospital return of spontaneous circulation (ROSC). Electrocardiogram (ECG) suggested a possible coronary artery occlusion. Given the circumstances of the arrest, computed tomography (CT) of the head was performed which demonstrated a large subarachnoid hemorrhage (SAH). Emergent percutaneous coronary intervention (PCI) was deferred due to the alternative explanation for the ECG changes and the patient’s instability. Her condition declined, and soon after the patient expired.

Patients with sudden cardiac arrest (SCA) due to SAH are unlikely to benefit from PCI, hindering a more appropriate workup and treatment.^1^ When faced with patients presenting with SCA and relevant risk factors, it is important to avoid anchoring bias and consider that the ischemic changes on ECG may not be due to primary cardiac causes.

**Topics:**

Subarachnoid hemorrhage, sudden cardiac arrest, pulseless electrical activity, ECG, CT.

**Figure f1-jetem-5-3-v18:**
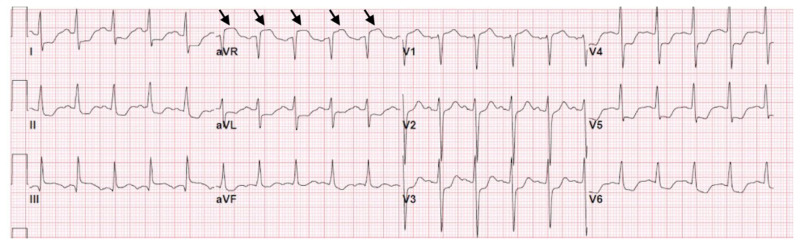


**Figure f2-jetem-5-3-v18:**
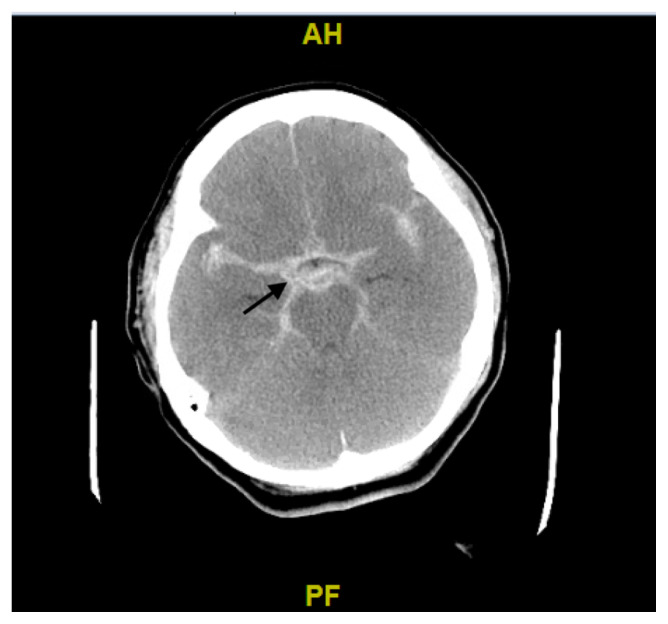


## Introduction

[Fig f1-jetem-5-3-v18][Fig f2-jetem-5-3-v18]Sudden cardiac arrest (SCA) is the sudden cessation of cardiac function leading to failure of circulation, respiration, and consciousness. The standard intervention for SCA is Advanced Cardiac Life Support (ACLS), involving chest compressions, effective ventilations, and hemodynamic stabilization. Evaluation for reversible causes is also performed, including gathering medical history, physical examination, and diagnostics. While the majority of SCA are caused by underlying cardiac disease and arrhythmias, other etiologies include: electrolyte disturbances, drug intoxication, autonomic nervous system dysfunction, and acute intracranial events.^2^

## Presenting concerns and clinical findings

A 40-year-old female with a past medical history of hypertension was brought in by ambulance to the ED as an unwitnessed full arrest with prehospital ROSC. The patient was showering after sexual intercourse when a family member heard the patient collapse in the bathroom. Emergency medical services (EMS) arrived and immediately initiated ACLS protocols. The cardiac monitor initially showed pulseless electrical activity (PEA). ROSC was achieved after 20 minutes of resuscitation with multiple rounds of intravenous epinephrine. The patient arrived to the ED, intubated, with a Glasgow Coma Scale (GCS) of 3 and fixed, dilated pupils.

## Patient Course

The patient presented to the ED after an episode of SCA and an ECG suggestive of possible myocardial infarction. The patient arrived intubated with a GCS of 3 and pupils fixed and dilated. Differentials including metabolic and toxicological etiologies were considered less likely given the patient’s history, clinical presentation, and labs results. Due to the ECG findings in the context of a full arrest, Code STEMI was activated. After discussing the case with interventional cardiology, the cardiac catheterization team was mobilized and preparations were made for emergent PCI; however, based on the post-coital presentation both the ED and cardiology teams agreed to rule out other etiologies for the SCA prior to going to the catheterization lab. Preparations were also made to start targeted temperature management due to the patient’s poor neurologic condition after ROSC. A computerized tomography (CT) of the head was ordered to evaluate for possible intracranial hemorrhage and non-contrast CT head showed evidence of a large SAH, which was suspected to be the cause of the patient’s cardiopulmonary arrest. PCI was deferred given the alternative diagnosis and concerns for the patient’s stability as the patient became hypotensive requiring multiple vasopressors. Due to concerns that the SAH was due to a ruptured berry aneurysm, neurointerventional radiology (NIR) evaluated the patient. She was ultimately considered a poor candidate for intervention due to a Hunt & Hess Grade V classification SAH (presenting with deep coma, decerebrate posturing, and moribund appearance with approximately 90% mortality) and hemodynamic instability.^3^ The patient was admitted to the intensive care unit (ICU), where she ultimately expired.

## Significant findings

The electrocardiogram demonstrated sinus tachycardia with ST segment elevation in lead aVR (black arrows) and diffuse ST depressions concerning for possible ST elevation myocardial infarction (STEMI). Given the events reported and the patient’s neurologic exam without sedation, non-contrast CT of the head was ordered; imaging showed evidence of a large subarachnoid hemorrhage, mostly at the level of the Circle of Willis (black arrow) concerning for an aneurysmal bleed as well as mild generalized white matter density suggestive of cerebral edema.

## Discussion

Aneurysmal subarachnoid hemorrhage (SAH) has an incidence of 7.9 per 100,000 person-years, and is typically caused by rupture of berry (saccular) aneurysms.^4^ Classic aneurysmal SAH is characterized by a sudden and severe headache, followed by loss of consciousness (LOC), nausea, vomiting, and meningeal irritation. Syncope and seizure are possible sequelae of SAH.^5^ Aneurysmal rupture can occur spontaneously but has also been associated with hypertensive emergencies, increased stress, straining, defecation, and sexual intercourse.^6^ Increases in blood pressure during intercourse may have had a role in this case. There is evidence that blood pressure may increase by 40–100 mm Hg systolic and 20–50 mm Hg diastolic during intercourse.^7^ In a study involving continuous monitoring of blood pressure during sexual activity in patients with hypertension, mean systolic pressures over 200 mm Hg in both males and females were reported.^8^ It is thought that a disruption in autoregulation of cerebral vasculature contributes to this phenomenon and may increase the risk for SAH.^7^ Non-traumatic SAH has a mortality rate of 50% with greater than 10% of patients expiring before arriving to the hospital, 25% within 24 hours of onset, and 45% within 30 days.^9^,^10^ Management of SAH involves blood pressure optimization, addressing coagulopathies if present, decreasing risk for vasospasm with nimodipine, and neurointerventional procedures as indicated.

Myocardial injury can arise after SAH leading to elevated troponin levels, ECG changes, and echocardiography abnormalities.^11^,^12^ Hypoperfusion of the posterior hypothalamus during SAH leads to the release of catecholamines, triggering coronary vasospasm.^13^ While it is suggested that this catecholamine release contributes to the myocardial injury associated with SAH, the etiology is multifactorial and not fully understood.^14^,^15^ Additionally, increases in intracranial pressure (ICP) associated with SAH can lead to loss of brainstem function resulting in respiratory arrest and subsequent hypoxia. Hypoxia causes tissue to release endogenous adenosine, which can decrease cardiac inotropy, slow atrioventricular conduction, and diminish pacemaker automaticity.^16^ Significant increases in ICP can also lead to a Cushing Reflex with bradycardia and irregular respirations that may lead to cardiac arrest.^17^ SAH can also present with a non-shockable cardiac rhythm such as asystole and PEA, as in this case.^10^,^18^

In this case, the patient presented after ROSC with an ECG that could be interpreted in different ways. ST segment elevation in aVR is associated with left main coronary artery (LMCA) disease, to the extent that it can be considered a STEMI equivalent warranting PCI.^15^,^16^ Alternatively, ST segment elevation in aVR with widespread ST segment depressions could represent global ischemia^19^, which may manifest alongside hypoxic brain injury after a period of cardiac arrest.^20^ In patients with post-arrest SAH, however, ST segment elevations can be seen on ECG despite finding normal coronary vessels on angiography, suggesting that emergent PCI in these patients may be futile.^1^,^2^ According to a study, evaluating patients with STEMI presentation on ECG being referred for primary PCI, there was a 2.3% incidence of non-acute coronary syndrome (ACS) cases.^21^ Thus it is imperative to retain a broad differential and high clinical suspicion of non-ACS conditions to avoid anchoring bias.

## Supplementary Information




