# Supplementary figures and images for: Post-Coital Sudden Cardiac Arrest Due to Non-Traumatic Subarachnoid Hemorrhage—A Case Report
Source: J Educ Teach Emerg Med. 2020 Jul 15;5(3):V18–21. doi: 10.21980/J8663N (PMC10332552; doi:10.21980/J8663N)

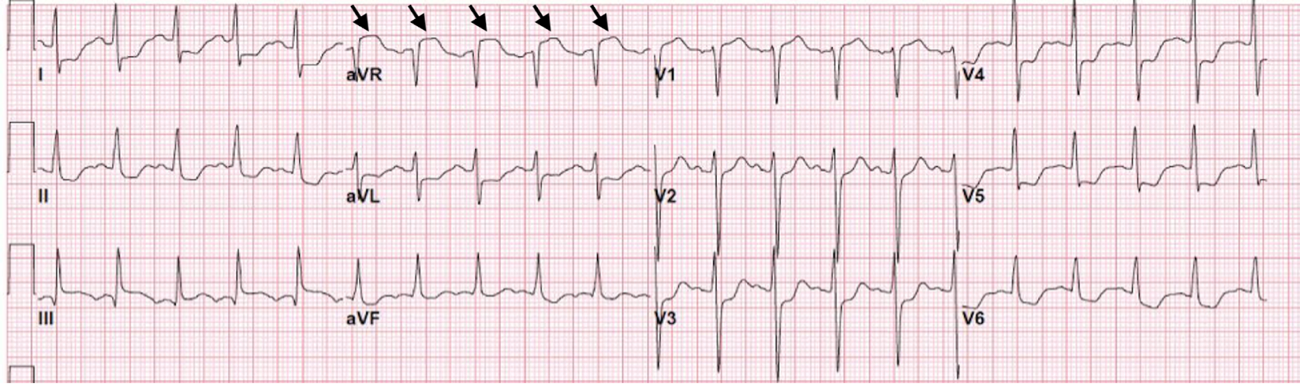

Supplement: Supplementary file 1 [file jetem-5-3-v18-supp1.png]

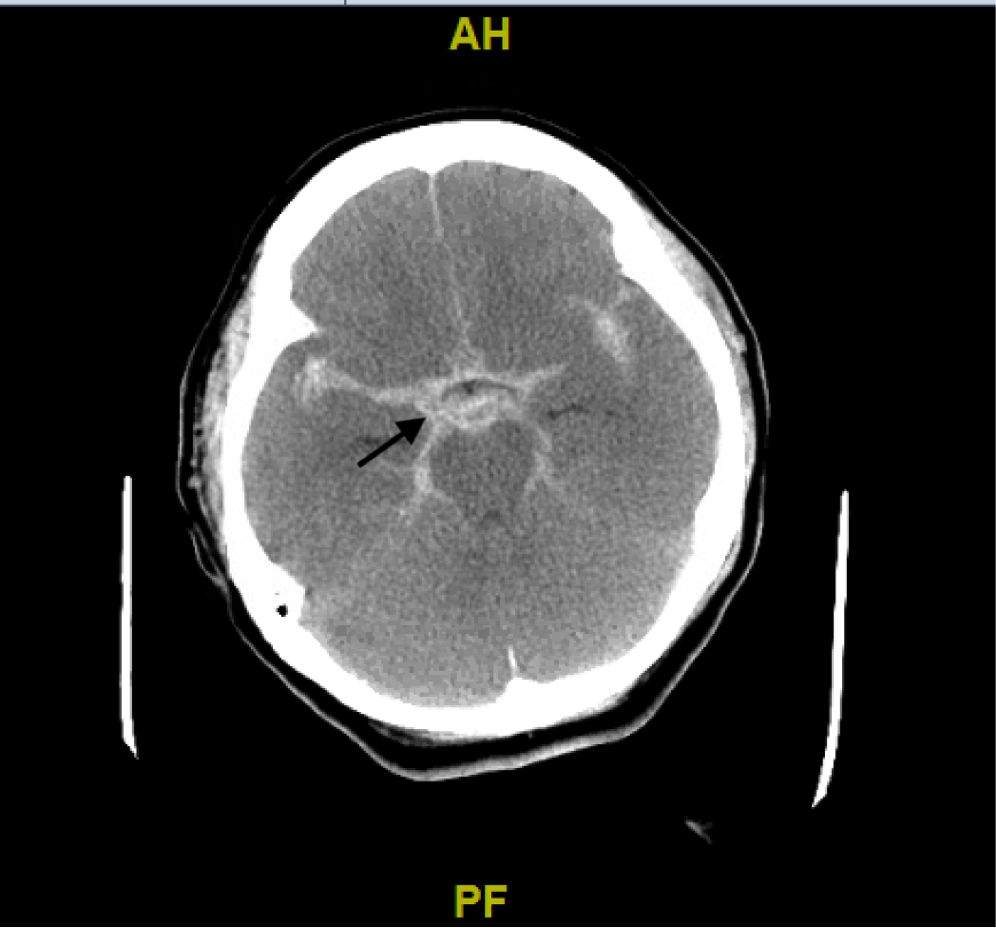

Supplement: Supplementary file 2 [file jetem-5-3-v18-supp2.png]
